# A versatile reporter system for CRISPR-mediated chromosomal rearrangements

**DOI:** 10.1186/s13059-015-0680-7

**Published:** 2015-05-28

**Authors:** Yingxiang Li, Angela I. Park, Haiwei Mou, Cansu Colpan, Aizhan Bizhanova, Elliot Akama-Garren, Nik Joshi, Eric A. Hendrickson, David Feldser, Hao Yin, Daniel G. Anderson, Tyler Jacks, Zhiping Weng, Wen Xue

**Affiliations:** Department of Bioinformatics, School of Life Science and Technology, Tongji University, Shanghai, P. R. China; RNA Therapeutics Institute and Program in Molecular Medicine, University of Massachusetts Medical School, Worcester, MA 01605 USA; David H. Koch Institute for Integrative Cancer Research, Massachusetts Institute of Technology, Cambridge, MA 02142 USA; Department of Biochemistry, Molecular Biology and Biophysics, University of Minnesota Medical School, Minneapolis, MN 55455 USA; Abramson Family Cancer Research Institute, University of Pennsylvania, Perelman School of Medicine, Philadelphia, PA 19104 USA; Department of Chemical Engineering, Massachusetts Institute of Technology, Cambridge, MA 02142 USA; Harvard-MIT Division of Health Sciences & Technology, Cambridge, MA 02139 USA; Institute of Medical Engineering and Science, Massachusetts Institute of Technology, Cambridge, MA 02142 USA; Program in Bioinformatics and Integrative Biology, University of Massachusetts Medical School, Worcester, MA 01605 USA

## Abstract

Although chromosomal deletions and inversions are important in cancer, conventional methods for detecting DNA rearrangements require laborious indirect assays. Here we develop fluorescent reporters to rapidly quantify CRISPR/Cas9-mediated deletions and inversions. We find that inversion depends on the non-homologous end-joining enzyme LIG4. We also engineer deletions and inversions for a 50 kb *Pten* genomic region in mouse liver. We discover diverse yet sequence-specific indels at the rearrangement fusion sites. Moreover, we detect Cas9 cleavage at the fourth nucleotide on the non-complementary strand, leading to staggered instead of blunt DNA breaks. These reporters allow mechanisms of chromosomal rearrangements to be investigated.

**Electronic supplementary material:**

The online version of this article (doi:10.1186/s13059-015-0680-7) contains supplementary material, which is available to authorized users.

## Background

Chromosomal deletions and inversions are common in human cancers, suggesting their causal roles in tumorigenesis [1]. In the past 2 years, the bacterial CRISPR [2] system has been transformed into a remarkable genome-editing tool [2–8]. The development of single-guide RNAs (sgRNAs) [7] allows the Cas9 nuclease to be readily targeted to specific genomic sequences with a downstream protospacer-adjacent motif (PAM), where Cas9 generates double-stranded DNA breaks that promote non-homologous end-joining (NHEJ) or homology-directed repair (HDR). NHEJ can result in indels that potentially inactivate the target gene and HDR generally results in precise DNA repair when guided by an exogenous donor molecule [6]. CRISPR/Cas9 genome editing tools have been successfully applied in many organisms, including mouse and human cells [9, 10]. We have recently applied CRISPR/Cas9 genome editing to repair a genetic disease gene [11] and study cancer drivers in the mouse liver *in vivo* [12]. This approach allowed one to rapidly identify and validate new cancer driver genes and to model cancer mechanisms in mice [13–15].

Engineering chromosomal rearrangements using traditional Cre-LoxP methods is technically challenging and time consuming [16]. CRISPR/Cas9 can also be used to model chromosomal rearrangements. Recent studies were performed on cell lines [3, 17–25], ES cells [26], mouse zygotes [27, 28], and lung cancer mouse models [16, 29]; however, detecting chromosomal rearrangements requires a series of indirect assays such as polymerase chain reaction (PCR) in single cell clones, Sanger sequencing, and fluorescent *in situ* hybridization. These low throughput assays limit the investigation of mechanisms of chromosomal rearrangements. Herein, we developed a fluorescent reporter system for directly detecting CRISPR/Cas9-mediated DNA inversions and deletions. We demonstrated that CRISPR/Cas9 could induce both deletion and inversion events in cultured cells and for a 50 kb *Pten* genomic region in the liver of adult mice.

## Results

**Fig. 1:**
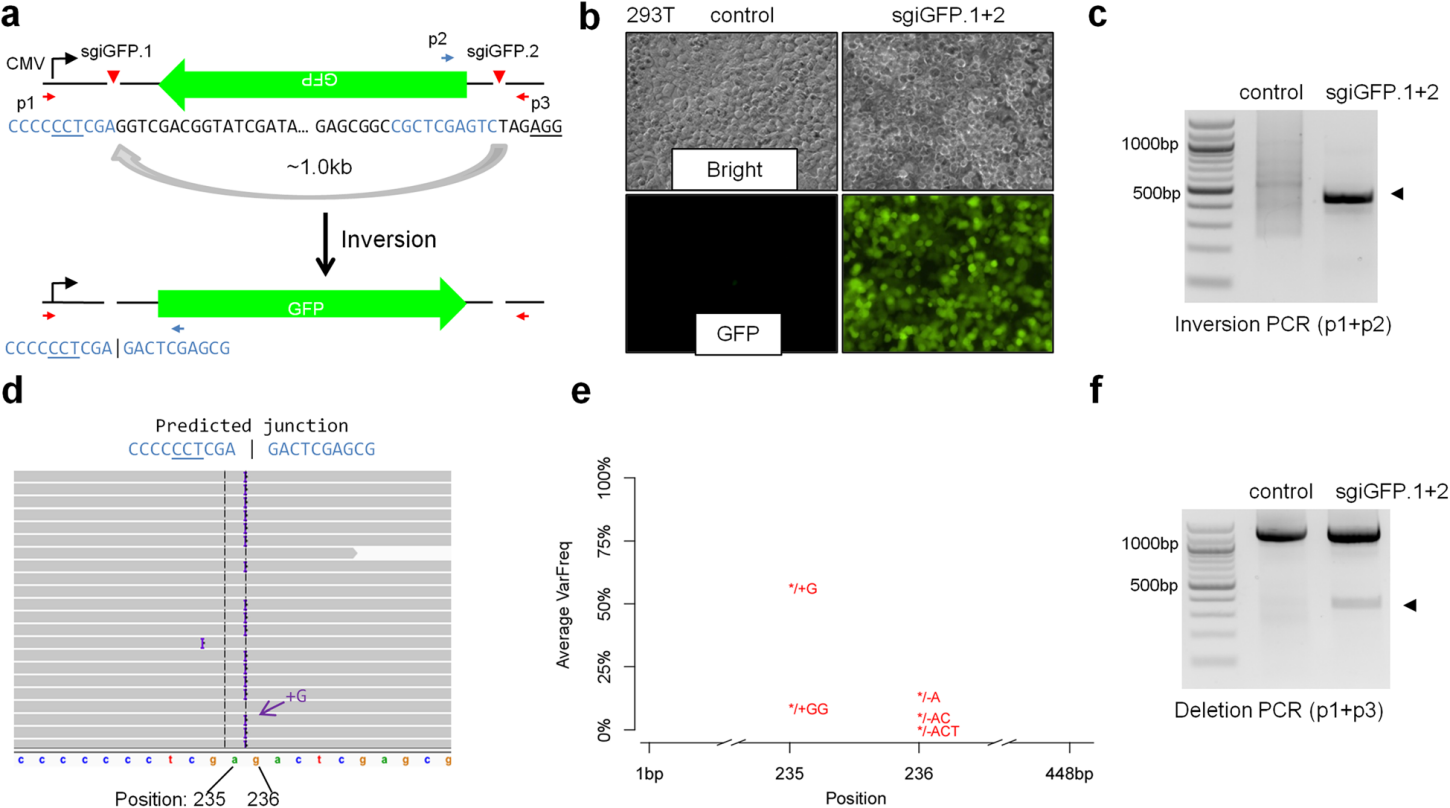
An inverted GFP reporter (iGFP) to visualize CRISPR/Cas9-mediated DNA inversion. **a** Schematic of iGFP. Red arrowheads indicate the Cas9 cutting sites recognized by the sgiGFP.1 and sgiGFP.2. Inversion of the GFP cassette will lead to GFP expression from the CMV promoter. PAM sequences are underlined. Red and blue color indicate sequences flanking the predicted fusion site (indicated by ‘|’). The blue sequence in the inverted plasmid will be reverse-complementary of the original sequence. **b** 293 T cells were co-transfected with 0.5 μg iGFP and 0.5 μg of two px330 plasmids (sgiGFP.1 + 2) and imaged 24 h later. **c** A PCR reaction detected inversion (primers p1 + p2) from total cellular DNA. The arrowhead indicates the expected inversion band. **d** Deep-sequencing identified perfect fusion and indels (insertions or deletions) at the DNA fusion sites. Purple bars in representative IGV images (two biological replicates) indicate insertions. Position indicates basepair position in the reference sequence. **e** Quantification of indels. VarFreq is the average of two replicates. 22 % of the reads mapped perfectly with predicted reference sequence, corresponding to precise ligation of the DNA breaks. **f** Two sgRNAs also induced deletion between CRISPR/Cas9 cutting sites. A PCR reaction detected deletion of the iGFP reporter (primers p1 + p3). The top bands are full length PCR products. An arrowhead indicates the expected deletion band

To develop a reporter system for visualizing chromosomal rearrangements, we used an inverted GFP (iGFP) plasmid [13] to mimic intra-chromosomal inversion (Fig. 1a). The GFP coding region was cloned in the inverted orientation after the cytomegalovirus (CMV) immediate-early promoter, preventing the expression of the GFP protein. We hypothesized that if we introduced two CRISPR/Cas9-mediated DNA breaks flanking the approximately 1.0 kb GFP cassette, we might be able to invert the orientation of the iGFP (Fig. 1a). We designed two sgRNAs targeting the flanking sequences (Fig. 1a and Additional file 1: Table S1). Co-transfection of two pX330 [30] plasmids co-expressing Cas9 and sgRNAs (hereafter named sgiGFP.1 + 2) with the iGFP plasmid in human 293T cells indeed led to GFP expression (Fig. 1b), confirming that cells can ligate distant DNA breaks from inverted DNA fragments [21].

To confirm that GFP expression was caused by inversion of the iGFP cassette, we designed PCR primers at the CMV promoter and the GFP N-terminal region, which could only amplify the inverted iGFP (Fig. 1a). PCR detected a band of the expected size in sgiGFP-transfected cells (Fig. 1c), suggesting that CRISPR/Cas9 can mediate DNA inversion between two sgRNA-directed cutting sites. To gain insights into how accurately these cells ligated the distant DNA breaks, we performed deep sequencing on the PCR band shown in Fig. 1c. We performed each experiment in two biological replicates, and obtained 1.2 and 0.6 M reads for the two replicates of sgiGFP.1 + 2 transfection, respectively. We predicted the reference sequence with an inverted iGFP, assuming that the Cas9 cutting site is 3 nucleotides (nt) upstream of the PAM (Additional file 2: Figure S1; see Additional file 1: Table S3 for reference sequences). We found that 96 % of the sequencing reads mapped to the reference (see Additional file 1: Table S4 for sequencing and mapping statistics). Thousands of reads mapped perfectly to the predicted fusion site, corresponding to a frequency of 22.2 % for precise ligation of the DNA breaks assuming that both of the cutting sites are 3 nt upstream of the PAM [2]. Other reads mapping to the fusion site revealed ‘+G’ (55.4 % frequency) and ‘+GG’ (7.5 %) insertions at position 235, as well as ‘–A’ (12.1 %) and other lower frequency deletions at position 236 of the reference (Fig. 1d, e and Table 1). The indel frequencies from the two biological replicates were in almost perfect agreement with each other (Table 1). We were surprised by the high frequencies of indels. Careful inspection of the sequences surrounding the cutting sites revealed that the ‘+G’ and ‘+GG’ insertions could have also been caused by alternative cutting sites of iGFP.1, that is, instead of cutting at 3 nt upstream of the ‘NGG’ PAM, these sequencing reads were consistent with cutting at 4 nt and 5 nt upstream of the PAM (Fig. 1a). It has been reported that Cas9 can cleave the complementary DNA strand at 3 nt and the non-complementary DNA strand within 3–8 nt upstream of the PAM, followed by trimming of the 3′ end by exonuclease activity [7]. Furthermore, if we assume that the cutting site of iGFP.1 is most frequently located at 4 nt upstream of the PAM, all the deletions can also be explained by the cutting sites of iGFP.2 being 4 nt, 5 nt, or 6 nt upstream of the PAM. Further studies are required to investigate whether CRISPR/Cas9 can induce DNA breaks at varying distances upstream of the PAM and contribute to repair of fusion sites.

**Table 1:** Summary of indels detected at the predicted inversion or deletion fusion sites

Name	Replicate	Position	Ref	Indel	Reads supporting ref	Reads supporting indel	VarFreq	*P* value
iGFP	1	235	A	+G	2631	4158	55.31 %	0
		236	G	-A	1786	257	11.83 %	7.64E-78
		235	A	+GG	2631	580	7.72 %	4.36E-181
		236	G	-AC	1786	50	2.30 %	1.67E-14
		236	G	-ACT	1786	35	1.61 %	4.61E-10
iGFP	2	235	A	+G	2662	4152	55.50 %	0
		236	G	-A	1852	276	12.40 %	6.55E-84
		235	A	+GG	2662	548	7.33 %	3.92E-170
		236	G	-AC	1852	35	1.57 %	4.63E-10
LoxP-O	1	304	A	+T	2278	4386	64.55 %	0
		305	T	-A	1590	42	2.51 %	3.88E-12
		302	T	-TA	7165	144	1.96 %	5.96E-35
		303	T	-A	6941	91	1.29 %	3.68E-20
LoxP-O	2	304	A	+T	2267	4429	64.89 %	0
		305	T	-A	1595	48	2.87 %	6.36E-14
		302	T	-TA	7186	140	1.90 %	8.19E-34
		303	T	-A	6974	82	1.16 %	9.96E-18
LSL	1	88	T	+A	1804	2533	56.19 %	0
		89	A	-T	1403	41	2.74 %	7.43E-12
		87	G	-T	4896	89	1.72 %	2.15E-22
		87	G	-TA	4896	87	1.68 %	8.00E-22
		86	C	-GTATAAT	5204	78	1.47 %	2.49E-18
		89	A	-TAAT	1403	15	1.00 %	2.51E-04
LSL	2	88	T	+A	1708	2602	58.47 %	0
		89	A	-T	1353	41	2.83 %	7.36E-12
		87	G	-TA	4820	95	1.85 %	4.07E-24
		87	G	-T	4820	87	1.69 %	7.96E-22
		86	C	-GTATAAT	5144	63	1.20 %	3.26E-14
		89	A	-TAAT	1353	17	1.17 %	6.93E-05
Pten-deletion	1	453	A	+C	6477	1110	14.16 %	0
		453	A	-T	6477	79	1.01 %	9.97E-18
Pten-deletion	2	453	A	+C	6607	1039	13.33 %	2.08E-314

CRISPR/Cas9 has also been implicated in mediating deletions between DNA break sites [21]. We designed PCR primers that could detect deletions between the two sgRNA sites in the iGFP reporter (Fig. 1a). A PCR reaction detected a lower band of expected deletion size only in 293T cells co-transfected with sgiGFP.1 + 2 (Fig. 1f), indicating a deletion of the iGFP reporter. These results confirmed that both inversion and deletion can occur between CRISPR/Cas9 cutting sites [21].

Because we used transfection of the iGFP reporter plasmids, our system may report iGFP inversion in a subset of plasmids, which could result from CRISPR/Cas9 interactions with weak PAMs. The iGFP plasmid also harbors two LoxP Orange (LoxP-O) sites for Cre recombinase-mediated inversion [13] (Additional file 2: Figure S2a). Because LoxP-O sites do not contain an ‘NGG’ PAM sequence, we designed a single sgRNA targeting a weak ‘NAG’ PAM [3] in the LoxP-O sites (see Additional file 1: Table S3 for sequences). Indeed sgRNA.LoxP-O induced GFP expression in 293T cells, albeit at a much lower level than sgiGFP.1 + 2 with the ‘NGG’ PAM (Additional file 2: Figure S2b). A PCR reaction detected a band of the expected size of sgiGFP in sgLoxP transfected cells (Additional file 2: Figure S2c). When we sequenced the PCR band, we observed that 64.7 % of fusion site mapping reads harbored a ‘T’ insertion (Additional file 2: Figure S2d, e; Table 1). Again, the insertion may be caused by the downstream cutting site being 4 nt upstream of the ‘AAG’ PAM.

**Fig. 2:**
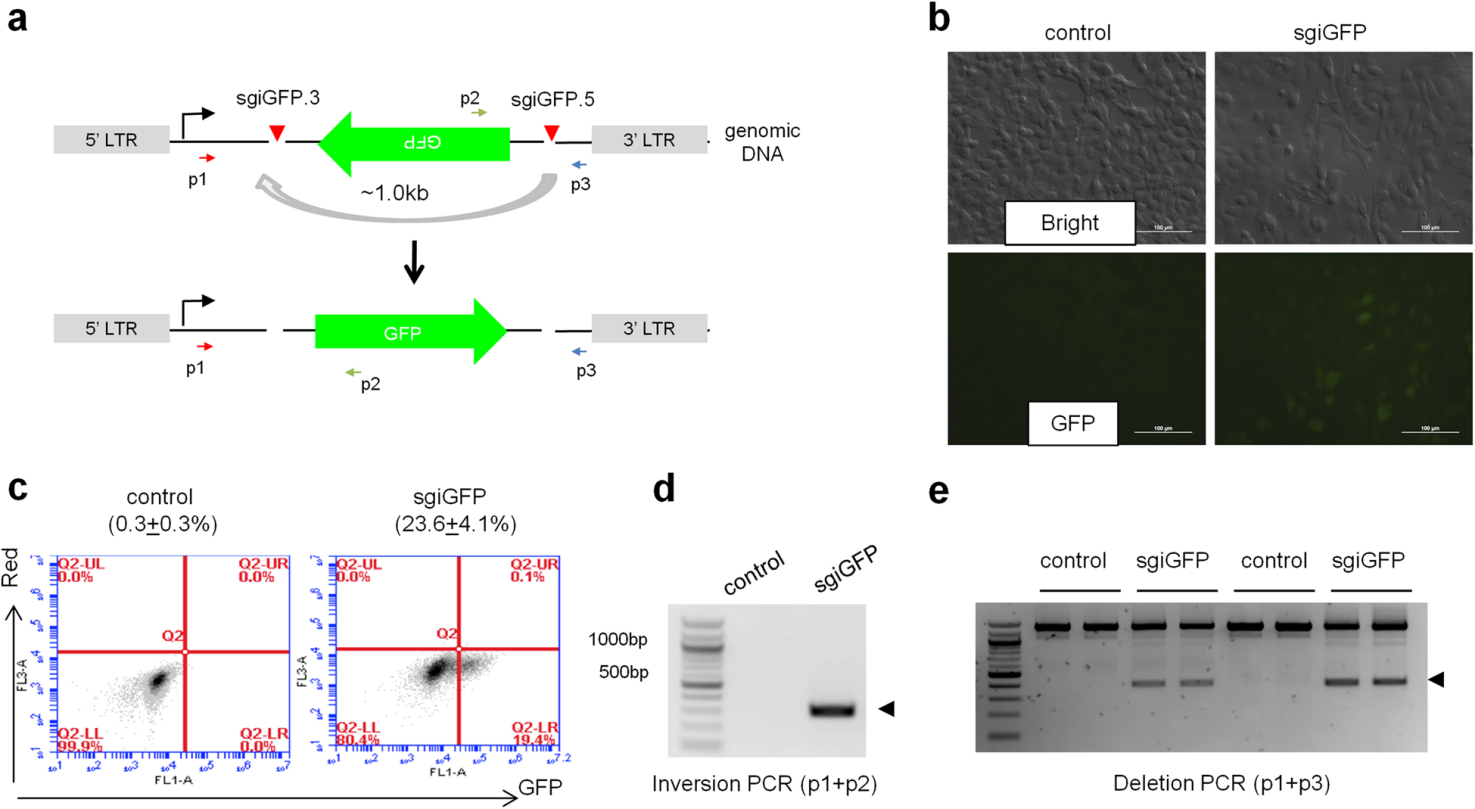
CRISPR/Cas9 mediates deletion and inversion of a chromosomal iGFP reporter. **a** Schematic of mouse cells harboring a chromosomal iGFP reporter. LTR is the long terminal repeat of the MSCV retroviral vector. Arrows denote PCR primers. **b** Cells were co-transfected with pX330 plasmids inversion sgiGFP.3 and sgiGFP.5 (sgiGFP) or control sgRNAs and imaged 72 h later. **c** FACS analysis to detect the population of GFP-positive cells. The averaged percentage of GFP^+^ cells is indicated (n = 3). **d** A PCR reaction detected inversion from genomic DNA. An arrowhead indicates the expected inverted band. **e** A PCR reaction detected deletion bands (arrowhead) from genomic DNA. The percentage of the deletion band intensity is 31.0 ± 7.4 % (n = 2)

We then used mouse 3T3 cells stably expressing a single copy of iGFP introduced via a retroviral vector to quantify iGFP inversion at a chromosomal locus [13] (Fig. 2a). Co-transfection of two sgRNAs sgiGFP.3 and sgiGFP.5 (hereafter named sgiGFP) targeting retroviral iGFP flanking sequences led to 23.6 ± 4.1 % GFP^+^ cells (Fig. 2b, c). Because the cells only had one copy of genomic iGFP, GFP^+^ cells were used to estimate the percentage of cells that underwent iGFP inversion. Importantly, our chromosomal iGFP reporter method offers a simple and fast assay to quantify cells with CRISPR-mediated genomic inversions and bypasses the laborious single cell cloning used in the literature [21]. In cells transfected with sgiGFP, PCR reactions detected bands of the expected sizes using primers that would detect inversion or deletion between sgRNA cutting sites (Fig. 2d, e), confirming that CRISPR/Cas9 can mediate both events [21]. By quantifying the deletion PCR bands (Fig. 2e), we estimated that the deletion PCR bands represent 31.0 ± 7.4 % of total PCR products. Although the 400 bp deletion PCR products presumably have higher PCR efficiency than the 1.4 kb full length PCR products, these numbers are consistent with a recent study showing approximately 30 % deletion efficiency of a 1.3 kb genomic region in mouse MEL cells [21].

**Fig. 3:**
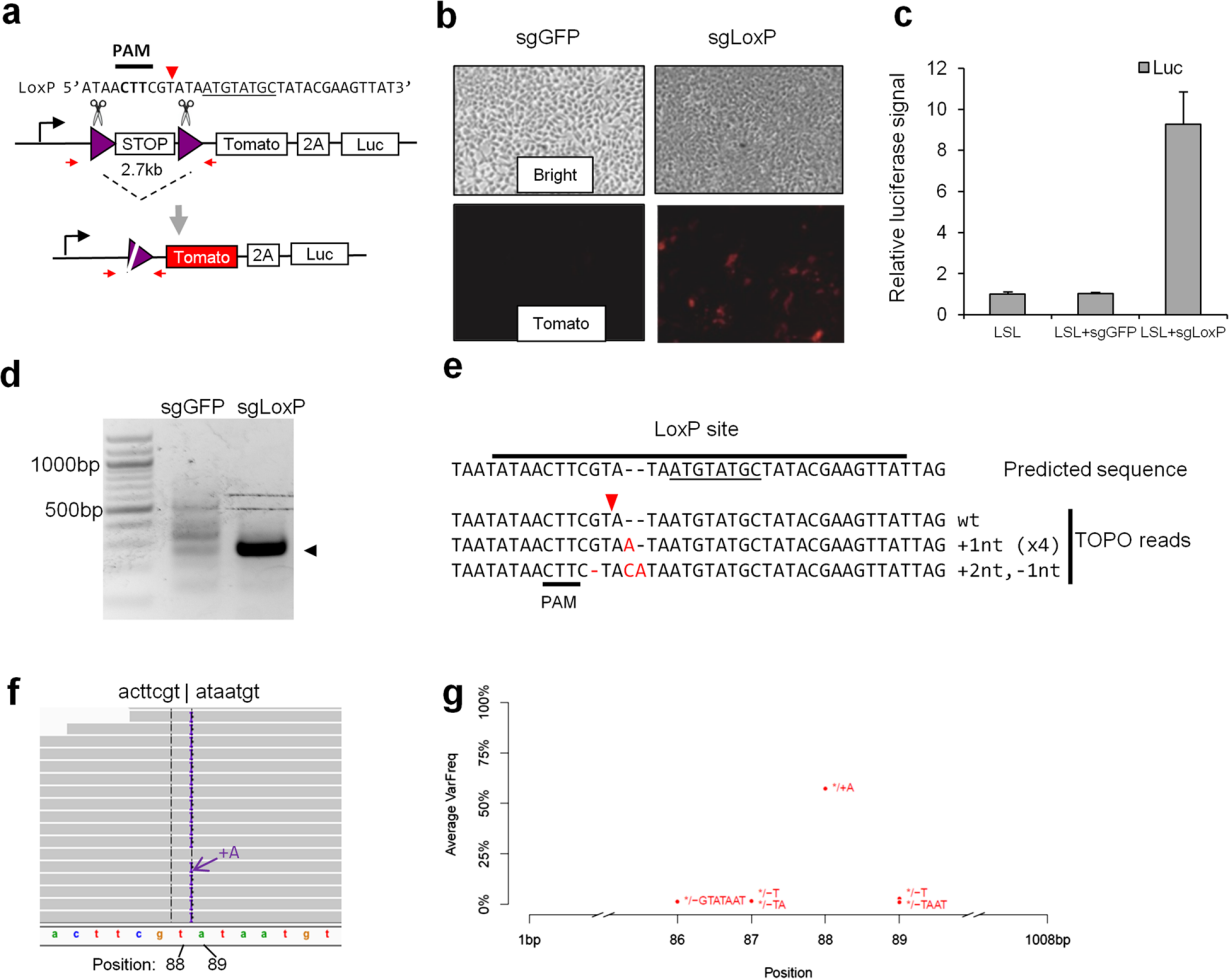
Modeling CRISPR/Cas9-mediated DNA deletion using a Lox-STOP-Lox (LSL) reporter. **a** Schematic of the LSL cassette (STOP is 2.7 kb) of a reporter plasmid (LSL). Purple triangles indicate the LoxP sites recognized by sgLoxP. The asymmetric 8 bp sequence of LoxP is underlined. The red arrowhead indicates the Cas9 cutting site. The red arrows indicate the location and direction of forward and reverse primers, respectively. The ‘NAG’ PAM in the LoxP sequence is in bold. **b** 293T cells were co-transfected with 0.3 μg LSL and 0.5 μg sgLoxP and imaged 48 h later. **c** The level of luciferase bioluminescence was quantified. Error bars are the standard deviation (s.d., n = 3). **d** A PCR reaction-detected deletion. An arrowhead indicates the expected deletion band. **e** PCR samples were purified, TOPO cloned, and sequenced. Red nucleotides indicate indels. **f** Deep sequencing. Representative IGV images of two biological replicates. **g** Count of indels

To monitor CRISPR/Cas9-mediated deletions, we developed a Lox-STOP-Lox reporter (hereafter named LSL), whereby an approximately 2.7 kb STOP cassette silences the transcription of a downstream tdTomato reporter, a self-cleaving 2A peptide, and luciferase (Fig. 3a). We designed a sgRNA to the LoxP sites with a weak ‘NAG’ PAM. Induction of both tdTomato and luciferase signals was observed in 293 T cells co-transfected with LSL and sgLoxP, indicating that the STOP cassette was removed by CRISPR/Cas9 (Fig. 3b, c). A PCR reaction confirmed that introduction of sgLoxP led to deletion between the LoxP sites (Fig. 3d). We performed TOPO cloning and Sanger sequencing on the deletion PCR band in Fig. 3d and detected error-free fusion and fusions with small indels (Fig. 3e). We further performed deep sequencing on the deletion PCR band in two biological replicates (Fig. 3f, g). Sequencing reads revealed ‘+A’ (57.3 %), ‘–T’ (2.8 %), and other lower frequency indels, along with a 34 % frequency for error-free fusion. Both ‘+A’ and ‘–T’ indels could be due to either NHEJ-mediated indels or by one cutting site being 4 nt upstream of the PAM. These results suggest that the CRISPR/Cas9 system can mimic the Cre recombinase in deleting sequences between LoxP sites, and that we have established a platform for monitoring CRISPR/Cas9-mediated deletions.

**Fig. 4:**
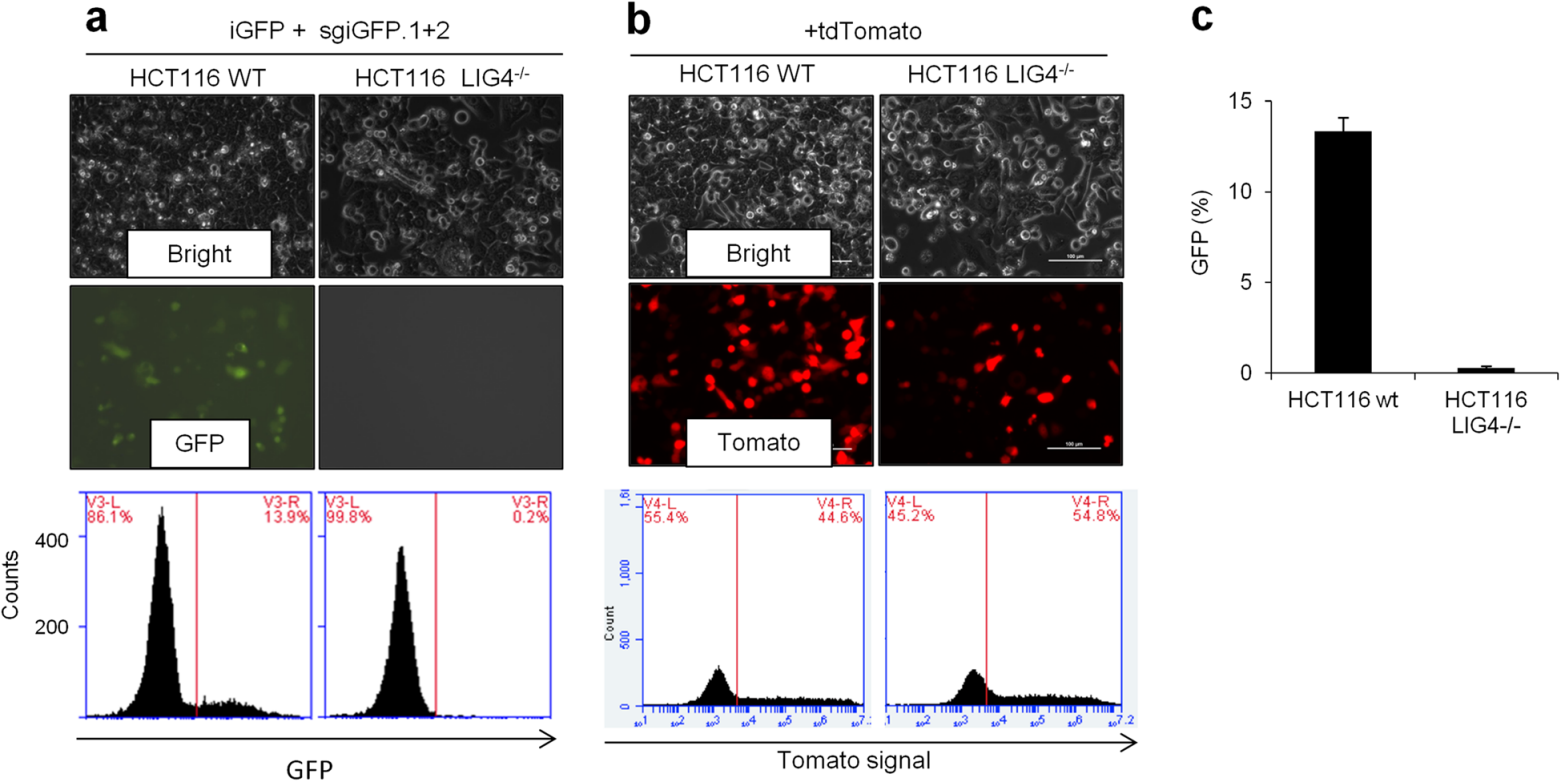
iGFP inversion is LIG4-dependent in human cells. **a** HCT116 wildtype (WT) or HCT116 LIG4^−/−^ cells were co-transfected with iGFP reporter and sgiGFP.1 + 2. Top: microscopic images. Bottom: FACS analysis of GFP positive cells. **b** Equal transfection efficiency in HCT116 WT and LIG4^−/−^ cells transfected with tdTomato plasmid. **c** Quantification of GFP^+^ cells in (a). Error bars are the s.d. (n = 3)

Our fluorescent reporters allow rapid detection of DNA rearrangement events, which could facilitate studying mechanisms of CRISPR-mediated DNA rearrangements in cells with defined mutations in DNA repair pathways. The non-homologous end-joining (NHEJ) pathway has been implicated in the fusion of DNA breaks to generate chromosomal rearrangements [31]. We asked whether this pathway was required for the repair of CRISPR/Cas9-mediated inversions. We used *LIG4*^−/−^ HCT116 cells [31] that are deficient in canonical NHEJ and severely impaired in chromosomal translocations. *LIG4* (Ligase IV) encodes a DNA ligase that joins double-strand DNA breaks during NHEJ [31]. Upon co-transfection of iGFP and sgRNA.1 + 2, we observed that GFP inversion was abolished in LIG4^−/−^ cells compared to LIG4 wild type cells (13.3 ± 0.8 % GFP in wild type HCT116 cells and 0.3 ± 0.1 % GFP in LIG4^−/−^ HCT116 cells, p = 4 × 10^−6^, Fig. 4a, c) at equal transfection efficiency (Fig. 4b). These results suggest that CRISPR/Cas9-mediated inversion is LIG4-dependent in human cells.

**Fig. 5:**
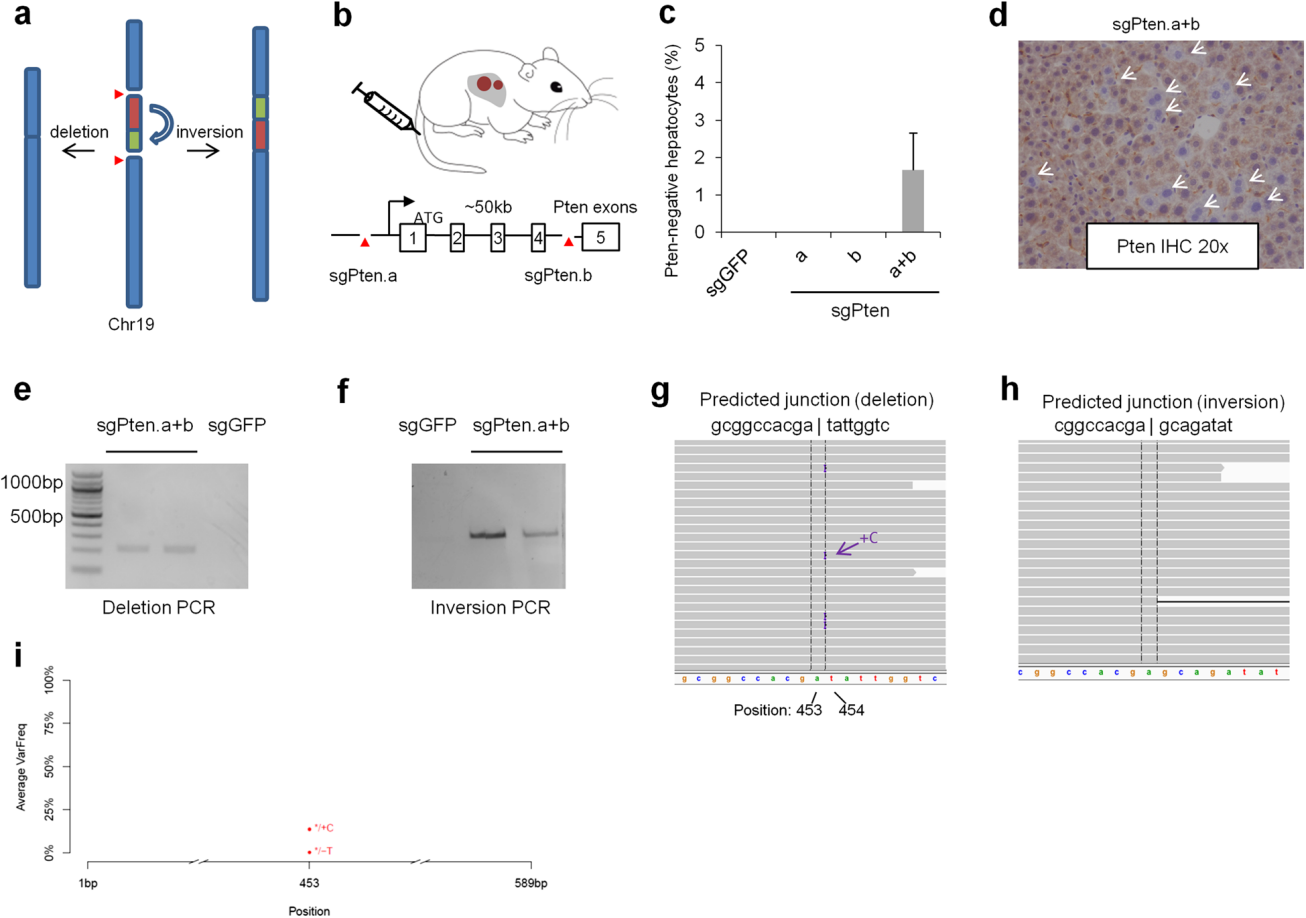
CRISPR/Cas9-mediated deletion and inversion of the *Pten* genomic region in mouse liver. **a** Schematic of deletion or inversion of a 50 kb Pten region on mouse chromosome 19. **b** Two pX330 plasmids expressing Cas9 and sgRNAs (sgPten.a + b) were co-delivered to mice via hydrodynamic injection. Red triangles indicate the sites recognized by sgPten.a and sgPten.b. The black arrow denotes the promoter. Liver tissue was analyzed 2 weeks later. **c** Quantification of Pten immunohistochemistry (n = 5 mice). Error bars are the s.d. **d** Pten-negative hepatocytes (arrows) were detected via immunohistochemistry. **e**, **f** A PCR reaction detected deletion (e) or inversion (f) of the targeted Pten region. **g**, **h** Deep sequencing of PCR bands detected approximately 14 % indel at the predicted deletion re-ligation site. We did not detect any indels with >1 % frequency at the predicted inversion repair site. Shown are representative IGV images of two biological replicates. **i** Quantification of deletion indels in **(g)**

Our cell culture data suggest that CRISPR can mediate both chromosomal deletions and inversions (Figs. 1 and 2). To explore whether CRISPR can mediate both events *in vivo*, we targeted a 50 kb region of the mouse genome encompassing the first four exons of the *Pten* [32] gene, a region frequently deleted in liver cancer [33] (Fig. 5a, b). We co-delivered two pX330 plasmids with two sgRNAs targeting non-coding *Pten* regions (sgPten.a + b) to five adult FVB mice by hydrodynamic tail vein injection [12], a method that delivers DNA to hepatocytes for transient expression. As controls, we injected an sgRNA targeting GFP (sgGFP), sgPten.a alone or sgPten.b alone in five mice per group. Two weeks later, immunohistochemistry identified hepatocytes with negative *Pten* staining in liver sections in sgPten.a + b mice but not in sgGFP, sgPten.a alone or sgPten.b alone groups (Fig. 5c, d and Additional file 2: Figure S3). Using PCR primers to detect chromosomal rearrangements (Fig. 5a), we observed deletion and inversion PCR products between the sgRNA target sites in genomic DNA from sgPten.a + b mice but not from sgGFP mice (Fig. 5e, f). We performed deep sequencing on the deletion and inversion PCR products, each from two mice. Because the deletion PCR bands were relatively weak, we did not perform gel extraction prior to sequencing (we performed gel extraction for all other deep sequencing samples in this study). We obtained 1 M and 1.4 M reads for the two deletion samples, among which 54 % of the reads mapped to the predicted reference sequence with the deletion and 42 % reads mapped to the mouse genome (Additional file 1: Table S4). Reads that mapped to the fusion site revealed the most frequent indel to be ‘+C’ (approximately 13.8 %) with a very high frequency (85.7 %) of error-free fusion (Fig. 5g, i; Table 1). Moreover, the ‘+C’ insertion could be caused by the cutting site of sgPten.b being 4 nt upstream of the PAM. We also obtained high quality deep sequencing data for the inversion PCR bands (2.1 M and 1.9 M reads for two mice, of which 99 % mapped to the predicted reference sequence). Strikingly, almost all reads that mapped to the fusion site did so perfectly, and we did not detect any indel with greater than 1 % frequency (Fig. 5h).

Because CRISPR/Cas9 has known off-target effects [34], we measured the indel rates at the top four predicted off-target sites of sgPten.a. In mouse 3T3 cells transfected with sgPten.a, the surveyor nuclease assay detected indels at the on-target *Pten* site but not at any of the assayed off-target sites (Additional file 2: Figure S4). These results indicate that CRISPR/Cas9 can mediate chromosomal inversion and deletion in the mouse liver with high specificity.

**Fig. 6:**
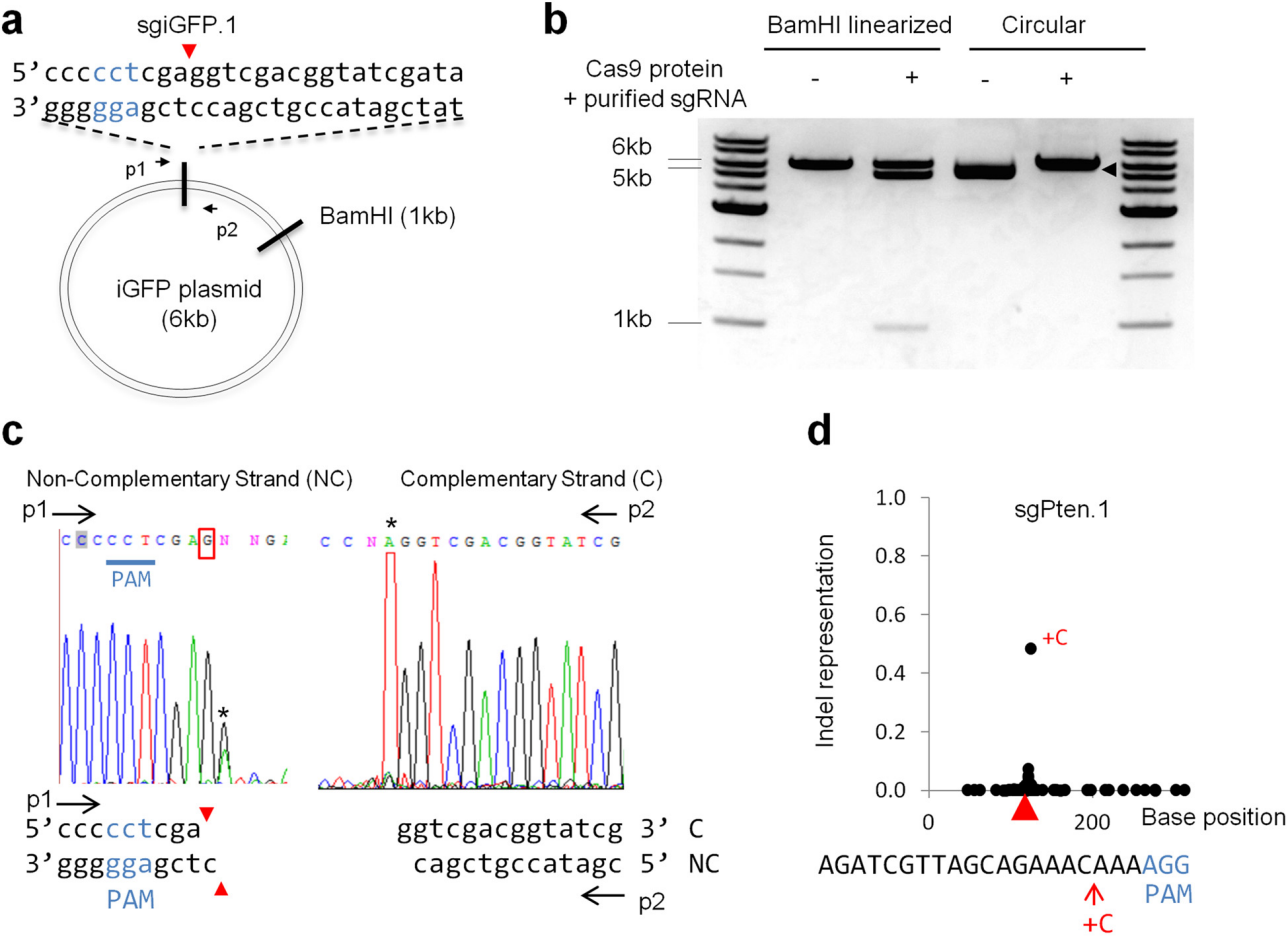
Cas9 can generate staggered DNA breaks. **a**–**c** Biochemical mapping identified non-canonical Cas9 cleavage sites. **a** Schematic of Cas9 cleavage assay. ‘cct’ is PAM. **b** *In vitro* cleavage of BamHI linearized or circular DNA by Cas9 protein and purified sgRNA. The expected cleavage products are 5 + 1 kb for linearizd iGFP plasmid. The size shift (arrowhead) of circular iGFP plasmid indicates Cas9 cleavage. **c** Sequencing analysis of cleaved products. Red arrowheads indicate Cas9 cleavages sites on two DNA strands. The 3′ terminal A or T (asterisks), caused by artifacts of sequencing reactions, indicate termination of primer extension and the position of the Cas9 cleavage sites [7]. The circled ‘G’ in sequencing trace indicates that Cas9 can cut at fourth nt on the non-complementary strand. The downstream weak ‘G’ peak overlapping with ‘A’ implies fifth nt cleavage. **d** Fourth nucleotide insertion of ‘C’ nucleotide* (+C) was frequently observed at sgPten target site after single sgRNA transfection in mouse cells. Indel representation is the ratio of selected indel versus all observed indels. Arrowhead indicates predicted Cas9 target sites. The position of the most abundant insertion (red arrow) is indicated in the target sequence. PAM sequence is in blue

While the canonical view is that *S. pyogenes* Cas9 generates a blunt end at 3 nt upstream of PAM [6], our deep sequencing data suggest non-canonical Cas9 cleavage (Fig. 1e). To map the Cas9 cleavage site of sgiGFP1, we performed *in vitro* Cas9/sgRNA cleavage assay using Cas9 protein and *in vitro* T7 transcribed sgiGFP.1 RNA (Fig. 6a–c). By sequencing the ends of the cleaved iGFP plasmid, we observed that Cas9 cleaves the complementary DNA strand at 3rd nt (Fig. 6c). Interestingly, cleavage of Cas9 could occur at 4 nt upstream of PAM on the non-complementary strand (Fig. 6c and Additional file 2: Figure S5), suggesting that Cas9 can generate staggered DNA breaks with 1 nt 5′ overhang for some sgRNA. Our finding confirmed an earlier study [7] that Cas9 cuts at non-canonical positions (4–6 nt upstream of PAM instead of 3 nt upstream). Importantly, a fourth nucleotide insertion upstream of PAM is frequently observed at Cas9 target site in *Pten*, *p53*, and *Ctnnb1* genes in mouse cells following NHEJ (Fig. 6d and Additional file 2: Figure S6) [12], which is consistent with end filling and ligation of a staggered DNA break. This surprising feature of Cas9 cleavage can elucidate how CRISPR-mediated DNA breaks are repaired in cells. Further studies are required to investigate how non-canonical CRISPR/Cas9 cleavage contributes to DNA repair.

## Discussion

In summary, we have developed fluorescent reporter-based systems to quantitatively report CRISPR/Cas9-mediated DNA deletions and inversions. We have performed in-depth sequence characterization of the deletion and inversion breakpoints, shown the suitability of using a less favorable ‘NAG’ PAM to induce deletions, and shown a dependency for LIG4 in the CRISPR/Cas9-mediated inversions. A similar approach has recently been used to excise a mTmG (Tomato and GFP) two-color fluorescent Cre reporter allele [35]. These reporter systems can be used to identify the DNA repair enzymes required for the rearrangement and improve our understanding of the role of DNA repair pathways in genomic rearrangements. For example, our findings indicate that the NHEJ enzyme LIG4 is required for the CRISPR/Cas9-mediated inversion events; the first time that this enzyme has been implicated in inversion rearrangements outside of V(D)J recombination. The role of LIG4 in mediating the rearrangements and the application of CRISPR-Cas9 to induce inversions and deletions in cells and in mice have been reported by recent studies [16, 19, 21, 31].

We successfully deleted or inverted a 50 kb region in the mouse genome in a subset of hepatocytes, and although further experiments are required to test the upper size limit that can be accomplished by this technology, this observation is certainly encouraging. Future studies are also needed to characterize potential chromosomal rearrangements induced by off-target Cas9 cutting [36].

We observed that CRISPR/Cas9 cutting sites were either re-ligated perfectly or with small indels, which is concordant with recent studies using CRISPR/Cas9 to induce chromosomal rearrangements in cells [19, 21]. We tested three inversions and two deletions, each with two biological replicates. Even though the indels were dominated by one-nucleotide insertions, we observed the insertion of all four types of nucleotides. This can be explained by either NHEJ-induced errors or by one CRISPR/Cas9 cutting site being 4 nt upstream of the PAM, at least on one DNA strand. These results are particularly striking given the prevailing notion that the S. pyogenes Cas9 cutting site is almost invariably located at 3 nt upstream of the PAM [2]. In our study, the identity and frequency of the types of nucleotide being inserted are reproducible between biological replicates. Notably, the LSL deletion and LoxP-O inversion constructs used similar LoxP and LoxP-O sites (in reverse complement), and accordingly the indels we observed were similar (also in reverse complement) between the two constructs, despite one construct mediating deletions and the other construct mediating inversions (Additional file 1: Table S3). Thus, the identity and frequency indels are likely specified by the sequences at the cutting sites. It would be intriguing to study the molecular mechanism underlying this exquisite sequence specificity.

NAG PAMs have not been considered in many CRISPR off-target studies [34], whereas our assay detected significant editing for sgLoxP and sgLoxP-O ‘NAG’ sites using sensitive cellular reporters. Because of the much interest in generating targeted chromosomal rearrangements using CRISPR/Cas9, our observation of the suitability of using a less favorable ‘NAG’ PAM to induce deletions suggests the need for in-depth characterization of unwanted rearrangements between off-target sites with ‘NAG’ PAMs. Notably, some variants of LoxP site such as Lox71 do not contain the NAG PAM targeted by our sgRNA.

We tested three constructs (two inversions and one deletion) by transfecting them into cell lines and noted that the frequencies of error-free fusion were in the range of 22–34 %. We also deleted and inverted a 50 kb region of the *Pten* gene in mice, achieving 86 % and 100 % error-free fusions for deletion and inversion, respectively. It appears that the repair of CRISPR/Cas9-mediated inversion and deletion events are dependent on the specific genomic sequences and sgRNAs used.

## Conclusions

In summary, these fluorescent reporters can provide a new method to rapidly quantify CRISPR-mediated DNA rearrangements and underscore the importance of genome editing as a potential tool to study mechanisms of chromosomal rearrangements.

## Materials and methods

### CRISPR vectors

sgRNA oligos were annealed and cloned into the pX330 vector using a standard BbsI protocol (Additional file 1: Table S1).

### Purification of genomic DNA and the surveyor nuclease assay

Genomic DNA was purified from mouse liver using High Pure PCR Template Preparation Kit (Roche). For the surveyor nuclease assay, PCR products were purified with a QIAquick Gel Extraction Kit and treated with the Surveyor nuclease kit (Transgenomic). DNA was electrophoresed on a 4 % to 20 % Novex TBE Gel (Life Technologies) with ethidium bromide staining. PCR products of LoxP regions were cloned using Zero Blunt TOPO PCR Cloning Kits (Life Technologies) and sequenced by the Sanger method [12].

### Deep sequencing of CRISPR modified chromosomal rearrangements

Inverted or deleted DNA regions were PCR amplified using Herculase II high-fidelity polymerase and PCR purified using a QIAquick Gel Extraction Kit. Libraries were made from the PCR products using the Nextera XT protocol [12] and sequenced on Illumina MiSeq (250 bp paired-end). Data were processed according to standard Illumina sequencing analysis procedures [12]. Reads were mapped to the reference sequences from predicted genomic inversion or deletion events. Insertions and deletions were called using VarScan2. *Pten*, *p53*, and *Ctnnb1* indels were analyzed using published deep sequencing dataset [12].

### Cell culture and transfection

Cell culture conditions were as described [12]. A total of 293T and mouse cells were transfected in 24-well plates using Mirus LT1 or Lipo3000 reagents, respectively. HCT116 cells were transfected with Lipofectamine 2000. GFP images were acquired at 24 to 72 h (20X lens) and total cellular DNA was harvested using QuickExtract reagent. For luminescence assay, cells were incubated with 30 mg/mL luciferin at a 1:200 dilution and assayed using a Tecan plate reader. FACS was performed on an Accuri C6 Flow Cytometer (BD). All data are representative of at least two independent transfections.

### Animal experiments

All animal study protocols were approved by the University of Massachusetts institutional animal care and use committee. pX330.Pten.a and pX330.Pten.b DNA (30 μg each) were delivered to approximately 8 week-old female FVB/NJ mice (Jackson Laboratories) by hydrodynamic tail vein injection. Plasmid DNA were purified using the EndoFreeMaxi Kit (Qiagen). An equal amount of sgGFP or single Pten sgRNA was used as controls.

### Immunohistochemistry

Mice were humanely euthanized by CO_2_ asphyxiation. Livers were fixed in 4 % or 10 % formalin overnight and embedded in paraffin. Liver sections of 4 μm were stained with hematoxylin and eosin (H&E) or standard immunohistochemistry protocols using an anti-Pten antibody (Cell Signaling). The number of hepatocytes was quantified from >3 low-magnification fields per mouse with five mice per group.

### *In vitro* transcription of sgRNA

DNA templates carrying a T7 promoter was PCR-generated from pX330 plasmids using Herculase II Fusion DNA Polymerase (Agilent), ethanol precipitated, and *in vitro* transcribed with in house made rNTPs, T7 buffer, and T7 polymerase. After 2 h of incubation at 37 °C, 50 U of TurboDNase (Life Technologies) was added and incubated for 30 min at 37 °C. The reactions were stopped with half a volume of formamide loading buffer, and was followed by heat denaturation step for 5 min at 95 °C. Eight percent PAGE-Urea gel was prepared with SequaGel-Urea Gel system (National Diagnostics) and pre-run at 25 W. A total of 400 uL of each samples were loaded, run at 25 W for 1.5 h, visualized with UV lamp set on short wavelength, and gel purified.

### Plasmid DNA cleavage assay

Cas9 protein (NEB) and sgRNA were pre-incubated for 10 min at 37 °C according to NEB protocols. Circular or linearized DNA was added and incubated for 1 h. Samples were analyzed by 1 % agarose gel electrophoresis with ethidium bromide.

### Statistics

Student’s t-tests were used to determine *P* values.

## Additional files

Additional file 1: Table S1. sgRNA target site sequences. **Table S2.** Primer sequences. **Table S3.** Reference sequences. The sequences corresponding to the forward primers are underlined. Primers and amplicon length are indicated. **Table S4.** Mapping summary of deep sequencing data. Additional file 2: Figure S1. Schematic of CRISPR-mediated inversion and deletion. **Figure S2.** sgRNA targeting LoxP-O sites (sgLoxP-O) mediates inversion of iGFP reporter. **Figure S3.** Pten immunohistochemistry in control mice (n = 5). **Figure S4.** Assessing off-target cutting of sgPten.a. **Figure S5.** Biochemical mapping of Cas9 cleavage site for sgiGFP.2. **Figure S6.** Staggered Cas9 cleavage can influence NHEJ in mouse cells.

## Abbreviations

bp: base pair
CRISPR: clustered regularly interspaced short palindromic repeat
DSB: double-stranded break
GFP: green fluorescent protein
iGFP: inverted GFP
LSL: Lox-STOP-Lox
NHEJ: non-homologous end-joining
nt: nucleotide
PCR: polymerase chain reaction
sgRNA: single guide RNA
