# Supplementary material for: A versatile reporter system for CRISPR-mediated chromosomal rearrangements
Source: Genome Biol. 2015 May 28;16(1):111. doi: 10.1186/s13059-015-0680-7 (PMC4465146; doi:10.1186/s13059-015-0680-7)
Supplement: Additional file 1: Table S1. — sgRNA target site sequences. Table S2. Primer sequences. Table S3. Reference sequences. The sequences corresponding to the forward primers are underlined. Primers and amplicon length are indicated. Table S4. Mapping summary of deep sequencing data. [file 13059_2015_680_MOESM1_ESM.docx]

**Table S1. sgRNA target site sequences.**

| **sgRNA ID** | **20nt sequence (5’ to 3’)** | **PAM** | **strand** |
| --- | --- | --- | --- |
| iGFP.1 | Gtatcgataccgtcgacctcg | AGG | - |
| iGFP.2 | gagcggccgctcgagtctag | AGG | + |
| iGFP.3 | gatatcgaattcctgcagcc | CGG | + |
| iGFP.5 | gttgatccataacttcgtat | AGG | - |
| LoxP-O | Gcgtatagtacacattatacg | AAG | - |
| LoxP | Gcgtatagcatacattatacg | AAG | - |
| Pten.a | Gtttcatagcggccacgaagt | GGG | + |
| Pten.b | gctgtagtaatatctgctat | TGG | - |
| GFP | gggcgaggagctgttcaccg | GGG | + |

**Table S2. Primer sequences.**

| **ID** | **Sequence (5’->3’)** | **Notes** |
| --- | --- | --- |
| pZ-F | cgtgctggttattgtgctgt |  |
| pZ-rtTA-R | gtacagggtaggctgctcaa | PCR LSL deletion with pZ-F |
| TdTomato-R | gcgcatgaactctttgatga | Deep seq LSL with pZ-F |
| CMV-F | cgcaaatgggcggtaggcgtg | Forward from CMV of iGFP |
| EGFP-R | cgtcgccgtccagctcgaccag | 5’ end of EGFP, reverse primer |
| iGFP-R1 | aggaaaggacagtgggagtg | PCR iGFP deletion with CMV-F |
| MSCV5 | cccttgaacctcctcgttcgacc | PCR iGFP inversion with EGFP-R |
| MSCV-Rev | cagcggggctgctaaagcgcatgc | PCR iGFP deletion with MSCV5 |
| Pten-F5 | gtcctcgaacatgcaaggtc | PCR deletion with R10 |
| Pten-R10 | caagcattgtgctcttcactccag |  |
| Pten-R6 | tccgtgaatagagtgcacct | PCR inversion with R10 Surveyor primer with F5 |
| Pten-1F | agcactctgcgaactgagctacat | Deep seq with F5 for inversion |
| Pten-F9 | ctgcttgtgtaaccagctcc | Deep seq with R10 for deletion |
| Pten.aOT1F | agtgtgggaatcgggatgtt | sgPten.a off-target site1 (OT1) |
| Pten.aOT1R | ttcaggcaaaacaggaaggc |  |
| Pten.aOT2F | tcctgacaccctagcctctc | sgPten.a off-target site2 (OT2) |
| Pten.aOT2R | acatgctgaaagggtcctct |  |
| Pten.aOT3F | tgggaaattgaggtgaggct | sgPten.a off-target site3 (OT3) |
| Pten.aOT3R | aaggtctctctcagccacac |  |
| Pten.aOT4F | gttgtcgatgggaagctgac | sgPten.a off-target site4 (OT4) |
| Pten.aOT4R | catggccttcgaagtactgc |  |

**Table S3. Reference sequences.** The sequences corresponding to the forward primers are underlined. Primers and amplicon length are indicated.

| >LSL pZF TdTomatoR 1008bp cgtgctggttattgtgctgtctcatcattttggcaaagaattggccgctctagaactagtggatccggaacccttaatataacttcgtataatgtatgctatacgaagttattaggtccctcgacctgcagcccaagctaggccaccatgtctagactggacaagagcaaagtcataaacggagctctggaattactcaatggtgtcggtatcgaaggcctgacgacaaggaaactcgctcaaaagctgggagttgagcagcctaccctgtactggcacgtgaagaacaagcgggccctgctcgatgccctgccaatcgagatgctggacaggcatcatacccacttctgccccctggaaggcgagtcatggcaagactttctgcggaacaacgccaagtcataccgctgtgctctcctctcacatcgcgacggggctaaagtgcatctcggcacccgcccaacagagaaacagtacgaaaccctggaaaatcagctcgcgttcctgtgtcagcaaggcttctccctggagaacgcactgtacgctctgtccgccgtgggccactttacactgggctgcgtattggaggaacaggagcatcaagtagcaaaagaggaaagagagacacctaccaccgattctatgcccccacttctgagacaagcaattgagctgttcgaccggcagggagccgaacctgccttccttttcggcctggaactaatcatatgtggcctggagaaacagctaaagtgcgaaagcggcgggccgaccgacgcccttgacgattttgacttagacatgctcccagccgatgcccttgacgactttgaccttgatatgctgcctgctgacgctcttgacgattttgaccttgacatgctccccgggggaagtggagctagggagggcagaggaagtcttctaacatgcggtgacgtggaggagaatcccggccctgctagaatggtgagcaagggcgaggaggtcatcaaagagttcatgcgc |
| --- |
| >iGFP CMVF EGFP 448bp cgcaaatgggcggtaggcgtgtacggtgggaggtctatataagcagagctctccctatcagtgatagagatctccctatcagtgatagagatcgtcgacgagctcgtttagtgaaccgtcagatcgcctggagacgccatccacgctgttttgacctccatagaagacaccgggaccgatccagcctccggactctagcgtttaaacttaagcttggtaccgggccccccctcgagactcgagcggccgctctagaactagtggatccataacttcgtataggataccttatacgaagttatctcagctagaagccataacttcgtatagtacacattatacgaagttatgtttactactacatgttactggcgcgccaccatggtgagcaagggcgaggagctgttcaccggggtggtgcccatcctggtcgagctggacggcgacg |
| >LoxP-O CMVF EGFP 413bp  Cgcaaatgggcggtaggcgtgtacggtgggaggtctatataagcagagctctccctatcagtgatagagatctccctatcagtgatagagatcgtcgacgagctcgtttagtgaaccgtcagatcgcctggagacgccatccacgctgttttgacctccatagaagacaccgggaccgatccagcctccggactctagcgtttaaacttaagcttggtaccgggccccccctcgaggtcgacggtatcgataagcttgatatcgaattcctgcagcccgggataacttcgtatagtacacattatacgaagttatgtttactactacatgttactggcgcgccaccatggtgagcaagggcgaggagctgttcaccggggtggtgcccatcctggtcgagctggacggcgacg |
| >Pten-deletion F9 R10 589bp ctgcttgtgtaaccagctccccaggcgctcggccccgacagcgctcctgcggacggctcgtggatgctattctctgctccgatccggcaagagaggggtccagcagaccacacgggagaaggaggcgggggcgatcacctaatagagcagaggggaccaagctcctgccccaggagcacacagataggggaatgggaatttggaaagttccccaactaggaccacacgtgacctcctcctgaaagtagttccgaccgcggctcatgtatccttccacctcgcctttgagccctcccaggcctgctcgccccgcccactcgctggctgcagcttccgaacgtcccatactccacacccgggctcagtaaccgggtcctcgaacatgcaaggtccgacagggtcagaacctggccatcgcgatccaattctgccgggttttcatagcggccacgatattggtcctgatagaagttactacttgacaagaggcctgggtgacgtgcatttggattcagttgtactgataggctatgacgtgttcccttcatgcacagattcatcctccctggagtgaagagcacaatgcttg |
| >Pten-inversion F5 1F 511bp gtcctcgaacatgcaaggtccgacagggtcagaacctggccatcgcgatccaattctgccgggttttcatagcggccacgagcagatattactacagcattgccagacatgctccgaagaaagtataattactaatagagtcaactatggaaaagactgacttttcctatcccataataaaattacccagtgacttgtttttaaaatggtctcacaaacattatatttccatatacatgagattaatcactcacagaaaaacatacaactgaagtaaagtttatagcaaattaagtttcagatttttttcacatgaattaagaatctaagatgtctttgttctctatagtcagagttaagtttttgaaggcaagatgctattatctagaagcaagacttccgttctatcactactggtttttatcttactagtatctgttttgattctatcctattgagatccttaaatcctggtatgaactctgggatgtagctcagttcgcagagtgct |
| PAM  loxP ataacttcgtataATGTATGCtatacgaagttat LoxP-O ataacttcgtataATGTGTACtatacgaagttat |

**Table S4. Mapping summary of deep sequencing data.**

| **Name** | **Total Reads** | **Reads Uniquely Mapped  to Reference** | **Reads Uniquely mapped to mm9** | **Percentage of Uniqually Mapped Reads** |
| --- | --- | --- | --- | --- |
| iGFP | 1,223,850 | 1,176,628 | n.a. | 96.14% |
| iGFP | 627,450 | 607,398 | n.a. | 96.80% |
| LoxP-O | 3,700,870 | 3,631,252 | n.a. | 98.12% |
| LoxP-O | 715,260 | 698,033 | n.a. | 97.59% |
| LSL | 554,176 | 520,052 | n.a. | 93.84% |
| LSL | 716,014 | 670,168 | n.a. | 93.60% |
| Pten-deletion | 1,001,684 | 528,444 | 381,625 | 90.85% |
| Pten- deletion | 1,377,628 | 750,784 | 493,643 | 90.33% |
| Pten-inversion | 2,088,578 | 2,060,647 | n.a. | 98.66% |
| Pten- inversion | 1,871,564 | 1,846,038 | n.a. | 98.64% |
