# Supplementary material for: A versatile reporter system for CRISPR-mediated chromosomal rearrangements
Source: Genome Biol. 2015 May 28;16(1):111. doi: 10.1186/s13059-015-0680-7 (PMC4465146; doi:10.1186/s13059-015-0680-7)
Supplement: Additional file 2: Figure S1. — Schematic of CRISPR-mediated inversion and deletion. Figure S2. sgRNA targeting LoxP-O sites (sgLoxP-O) mediates inversion of iGFP reporter. Figure S3. Pten immunohistochemistry in control mice (n = 5). Figure S4. Assessing off-target cutting of sgPten.a. Figure S5. Biochemical mapping of Cas9 cleavage site for sgiGFP.2. Figure S6. Staggered Cas9 cleavage can influence NHEJ in mouse cells. [file 13059_2015_680_MOESM2_ESM.pdf]

Fig. S1

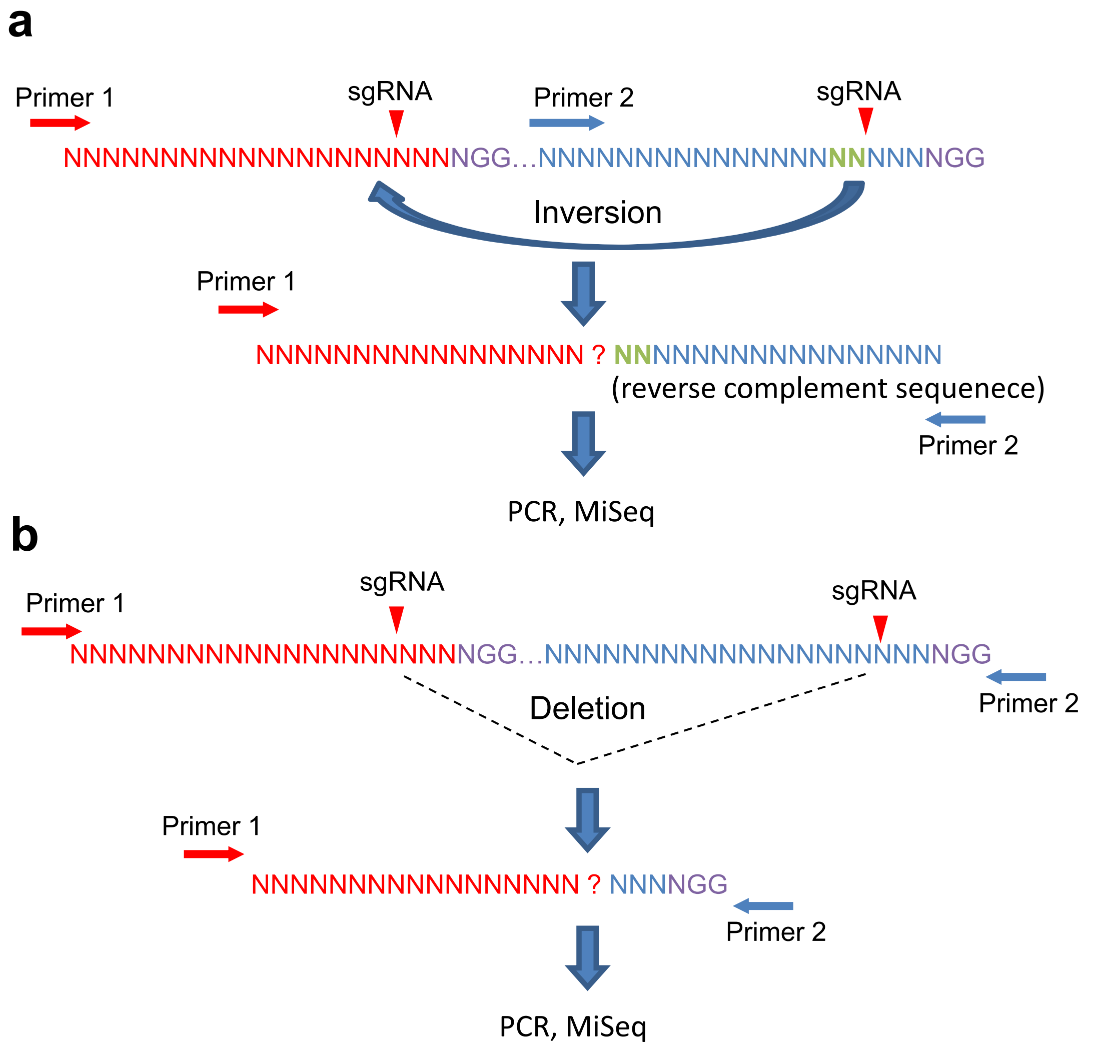

Figure S1. Schematic of CRISPR-mediated inversion and deletion. ? denotes potential indels at the fusion sites in inversion (a) or deletion (b) events.

Fig. S2

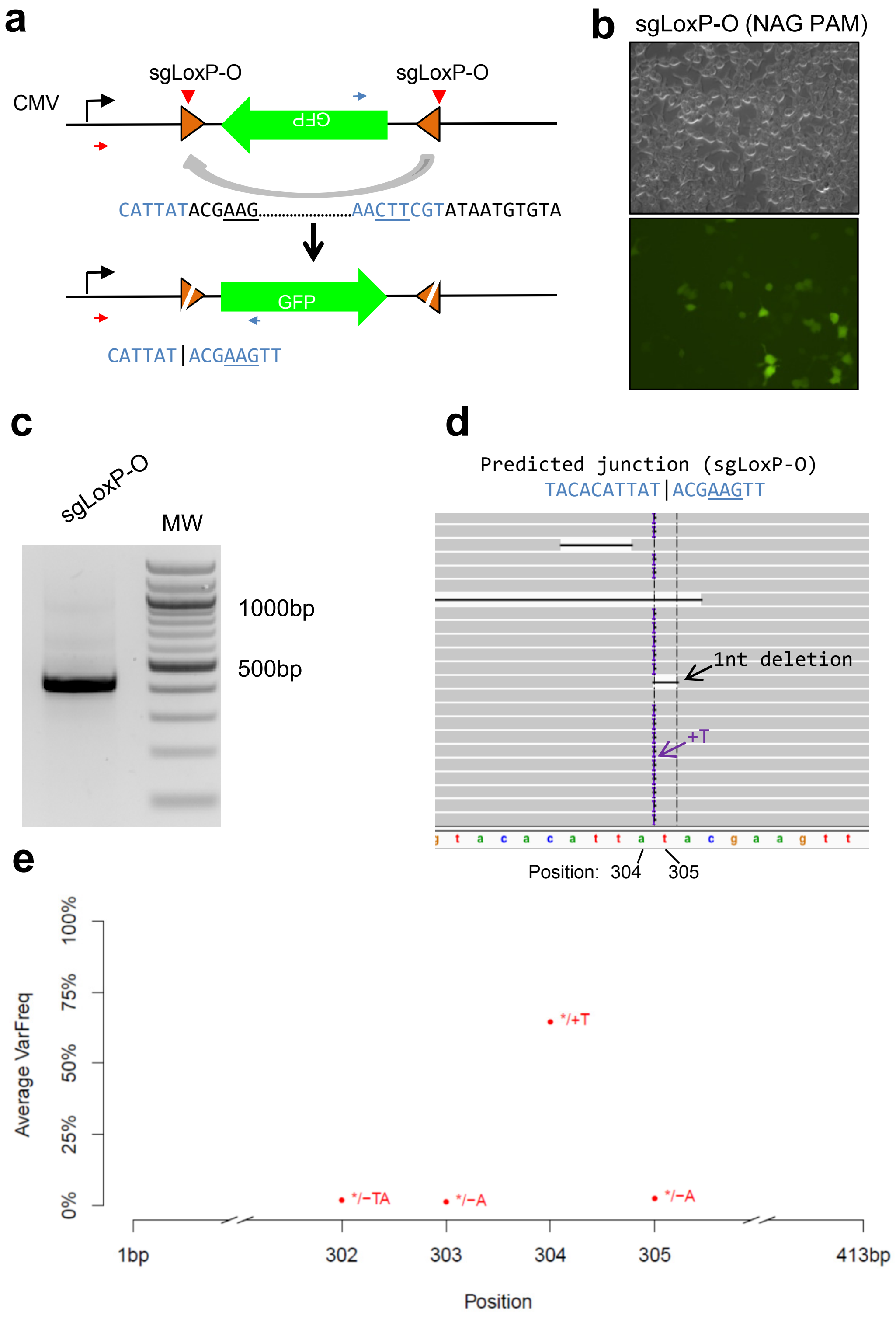

Figure S2. sgRNA targeting LoxP-O sites (sgLoxP-O) mediates inversion of iGFP reporter. (a) Orange triangles indicate the LoxP-O sites recognized by sgLoxP-O. Red arrow, forward primer. Blue arrow, reverse primer. White angled lines denote DNA fusion sites. (c) 293T cells were co-transfected with 0.5ug iGFP and 0.5ug pX330 plasmid expressing sgLoxP-O and imaged 24 hours later (20x). (c) PCR reaction detected inversion from genomic DNA. Arrowhead indicates the expected deletion band. (d) Deep-sequencing identified perfect fusion and indels at the DNA fusion sites. "|" in the predicted sequences denotes DNA fusion site. Black or purple bars in IGV images (two biological replicates) indicate deletions or insertions, respectively. (e) Quantification of indels.

Fig. S3

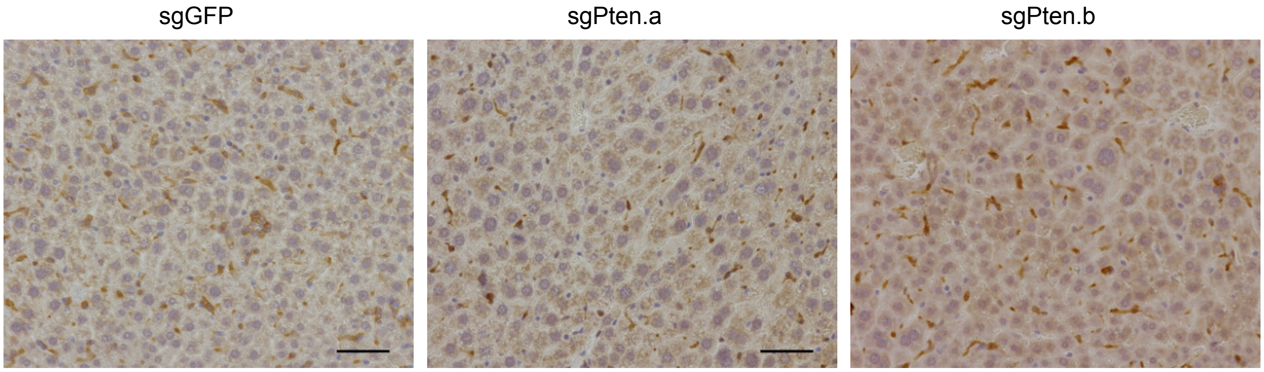

Figure S3. Pten immunohistochemistry in control mice (n=5). Scale bars are 50 μm.

Fig. S4

a

ID	Sequence (20nt+PAM)	score	mismatches	UCSC gene	locus
1	TCTCATTTCTGCCACGAAGTGAG	0.9	4MMs [2:7:8:10]		chr3:-53052171
2	TTTCCTTCCTGCCACGAAGTAGG	0.8	4MMs [5:7:8:10]		chr2:+68344380
3	TATGATAGCATCCACGAAGTCAG	0.7	4MMs [2:4:10:11]		chr15:+41452768
4	TTCCCTAGTGACCACGAAGTAGG	0.5	4MMs [3:5:9:11]	NM_173442	chr19:+17403966
5	TTTTCTAGAGGCCACGAAGACAG	0.4	4MMs [4:5:9:20]		chr12:-80711978
6	TATCAAAGCAGCCACGAAGAAAG	0.4	4MMs [2:6:10:20]		chr10:-10535645
7	TTCCAAAGCTGCCACGAAGGGGG	0.4	4MMs [3:6:10:20]	NM_010055	chr11:-94986011
8	TTTCCTAGCTACCACGAAGCAAG	0.3	4MMs [5:10:11:20]		chr3:+155933182
9	TTTCATAGCAGACAGGAAGTGAG	0.3	3MMs [10:12:15]		chr13:+14639997
10	CTTCCTAGCTGCCACGAGGTAAG	0.3	4MMs [1:5:10:18]		chr17:-5496239
11	TTTCTTAACAGCCACGAGGTGGG	0.3	4MMs [5:8:10:18]		chr10:-115658039
12	CTTCATTGCTGCCAAGAAGTGGG	0.3	4MMs [1:7:10:15]		chr12:-14028451
13	TTTGGTAGCGGCCACGGAGCCAG	0.3	4MMs [4:5:17:20]		chr12:+105232985
14	CTTCATATGGGCCAGGAAGTGAG	0.3	4MMs [1:8:9:15]		chr12:+55394979
15	TTATATAGTGGCCAAGAAGTCAG	0.2	4MMs [3:4:9:15]		chr2:-45928199
16	TTTAATACTGGCCAGGAAGTTGG	0.2	4MMs [4:8:9:15]		chr3:+18424178
17	TTTCATAGTGGCCAAGAAGAGAG	0.2	3MMs [9:15:20]		chr8:+118683236
18	CTTCATAGCAGGCAGGAAGTCAG	0.2	4MMs [1:10:12:15]		chr6:-86302227
19	TTTCTTGGCAGCCACGAGGTTAG	0.2	4MMs [5:7:10:18]		chr8:-28989612
20	CTTCATAGCTGCCATGAAGAGAG	0.2	4MMs [1:10:15:20]		chr13:+96356388

b

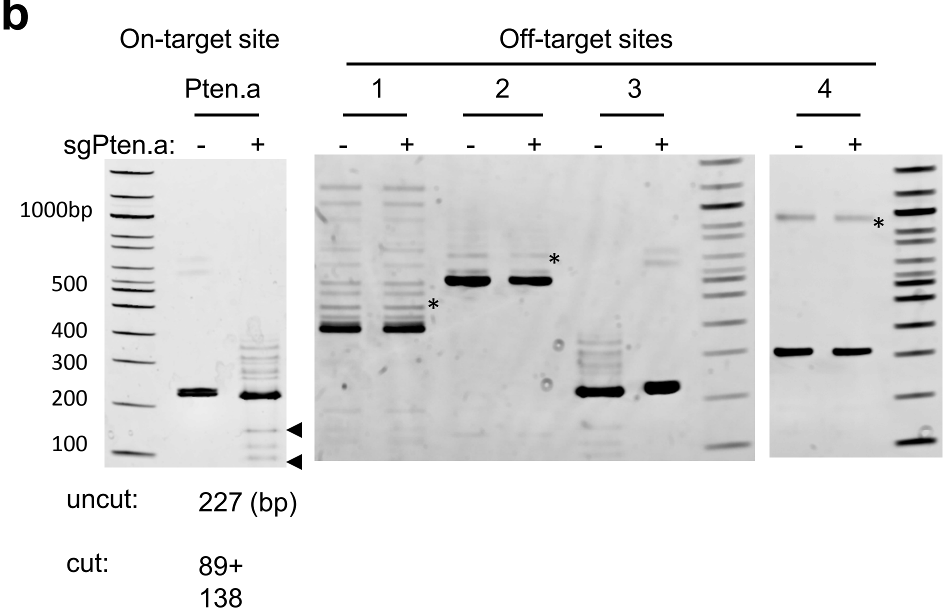

Figure S4. Assessing off-target cutting of sgPten.a. (a) Top 20 potential off-target sites in the mouse genome. Score is likelihood of off-target binding. (b) Surveyor assay in mouse 3T3 cells transfected with control (-) or sgPten.a (+). Predicted size of uncut and cut bands are indicated. Arrowheads denote surveyor nuclease cleaved fragments of the Pten PCR products. Asterisks denote non-specific bands.

Fig. S5

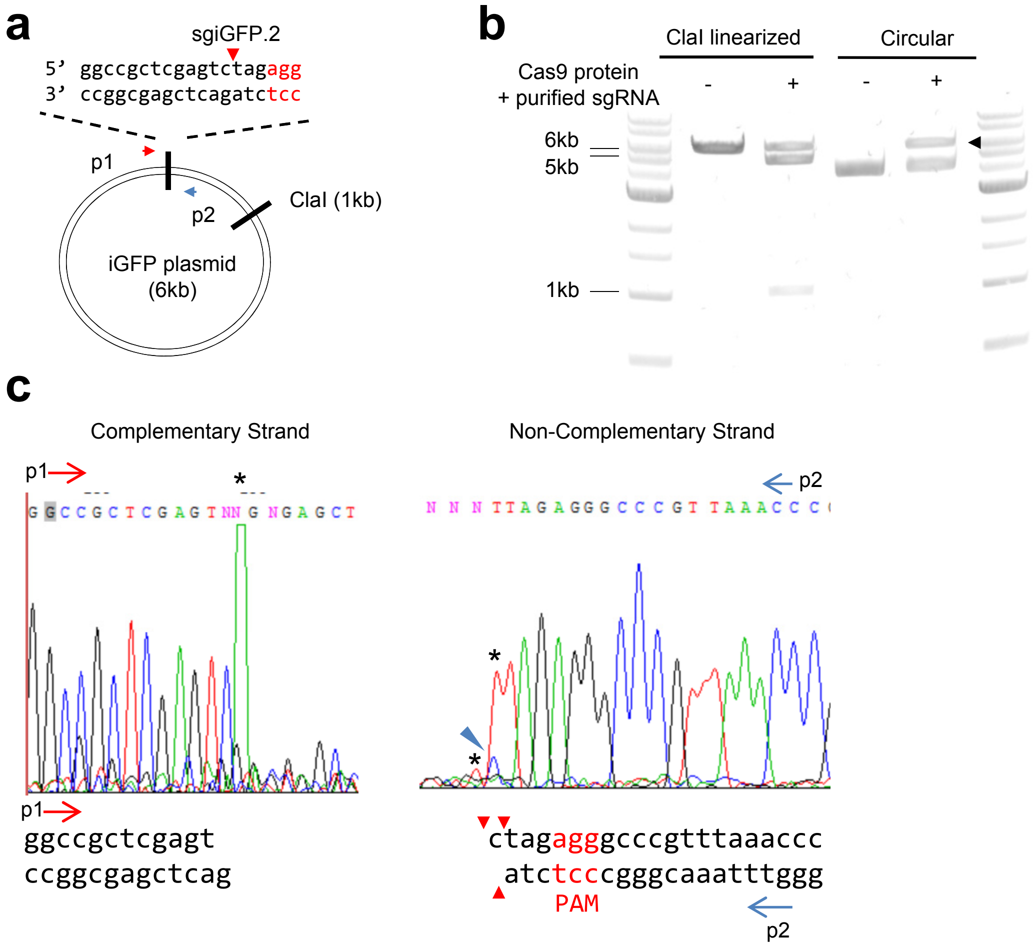

Figure S5. Biochemical mapping of Cas9 cleavage site for sgiGFP.2. (a) Schematic of Cas9 cleavage assay. "agg" is PAM. (b) *in vitro* cleavage of ClaI linearized or circular DNA by Cas9 protein and purified sgRNA. The expected cleavage products are 5+1kb for linearizd iGFP plasmid. The size shift (arrowhead) of circular iGFP plasmid indicates Cas9 cleavage. (c) Sequencing analysis of cleaved products. The 3' terminal A or T (asterisks), caused by artifacts of sequencing reactions, indicate termination of primer extension and the position of the Cas9 cleavage sites. Red arrowheads indicate Cas9 cleavages sites on two DNA strands. The small blue "C" peak (blue arrowhead) indicates 4thnt cleavage on the non-complementary strand in a subset of DNA molecules .

Fig. S6

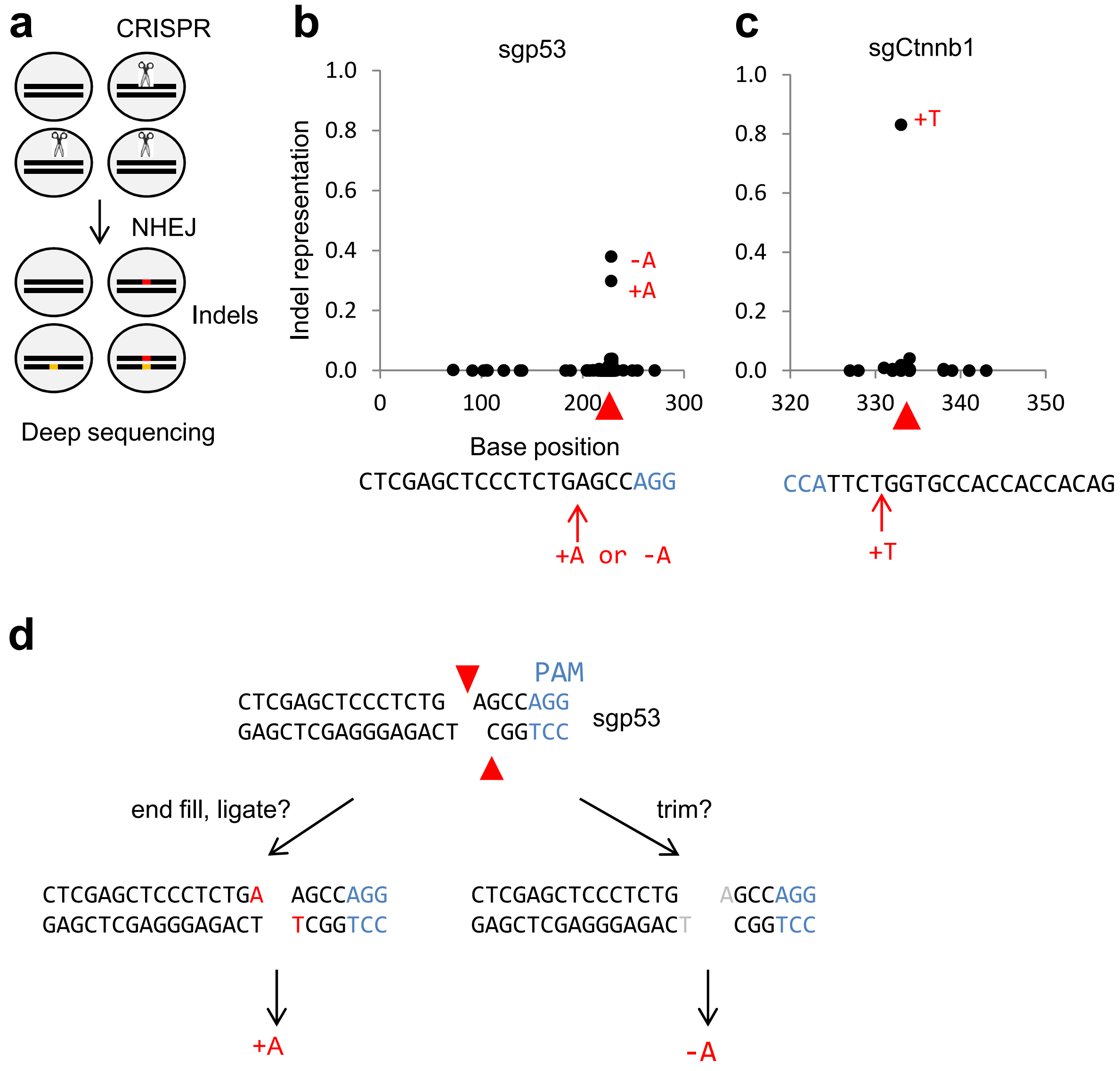

Figure S6. Staggered Cas9 cleavage can influence NHEJ in mouse cells. (a) A single sgRNA targeting protein coding region was introduced to mouse cells or liver. Indels at the target sites in cell population and liver tissue were quantified by deep sequencing. (b-c) 4th nucleotide insertion upstream of PAM is frequently observed at Cas9 target sites in mouse cells. Indel representation is the ratio of selected indel versus all observed indels. Arrowheads indicate predicted Cas9 target sites. The position of the most abundant insertion (red arrow) is indicated in the target sequence. PAM sequence is in blue. “+A” indicates A insertion. “-A” indicates A deletion. n=3 cell population and liver tissue for sgp53 and n=2 for sgCtnnb1. (d) A model linking staggered Cas9 cleavage with NHEJ-mediated 1nt insertion or 1nt deletion.
